# Lipid Extraction from* Spirulina* sp. and* Schizochytrium* sp. Using Supercritical CO_2_ with Methanol

**DOI:** 10.1155/2018/2720763

**Published:** 2018-12-09

**Authors:** Shihong Liu, Husam A. Abu Hajar, Guy Riefler, Ben J. Stuart

**Affiliations:** ^1^Department of Chemical and Biomolecular Engineering, Ohio University, Athens, OH, USA; ^2^Civil Engineering Department, School of Engineering, The University of Jordan, Amman, Jordan; ^3^Department of Civil Engineering, Ohio University, Athens, OH, USA; ^4^Department of Civil & Environmental Engineering, Old Dominion University, Norfolk, VA, USA

## Abstract

Microalgae are one of the most promising feedstocks for biodiesel production due to their high lipid content and easy farming. However, the extraction of lipids from microalgae is energy intensive and costly and involves the use of toxic organic solvents. Compared with organic solvent extraction, supercritical CO_2_ (SCCO_2_) has demonstrated advantages through lower toxicity and no solvent-liquid separation. Due to the nonpolar nature of SCCO_2_, polar organic solvents such as methanol may need to be added as a modifier in order to increase the extraction ability of SCCO_2_. In this paper, pilot scale lipid extraction using SCCO_2_ was studied on two microalgae species:* Spirulina *sp. and* Schizochytrium *sp. For each species, SCCO_2_ extraction was conducted on 200 g of biomass for 6 h. Methanol was added as a cosolvent in the extraction process based on a volume ratio of 4%. The results showed that adding methanol in SCCO_2_ increased the lipid extraction yield significantly for both species. Under an operating pressure of 4000 psi, the lipid extraction yields for* Spirulina *sp. and* Schizochytrium *sp. were increased by 80% and 72%, respectively. It was also found that a stepwise addition of methanol was more effective than a one-time addition. In comparison with Soxhlet extraction using methylene chloride/methanol (2:1, v/v), the methanol-SCCO_2_ extraction demonstrated its high effectiveness for lipid extraction. In addition, the methanol-SCCO_2_ system showed a high lipid extraction yield after increasing biomass loading fivefold, indicating good potential for scaling up this method. Finally, a kinetic study of the SCCO_2_ extraction process was conducted, and the results showed that methanol concentration in SCCO_2_ has the strongest influence on the lipid extraction yield.

## 1. Introduction

Fossil fuels, which include coal, petroleum, and natural gas, comprise 81% of the total energy consumed by mankind in 2014 [1]. Compared to other energy types, developing and producing fossil fuels is the most economical way of energy production. Also, the energy density of fossil fuels is above other alternative energy sources, which means fossil fuels are the most efficient energy sources. However, the use and consumption of fossil fuels has caused many problems. Because fossil fuels are considered to be a nonrenewable and nonsustainable form of energy, their production will decline and eventually be exhausted. Since the combustion of fossil fuels emits greenhouse gases (GHGs), the use of fossil fuels is a major contributor to global warming [2]. The extraction of fossil fuels also causes other forms of environmental degradation [3]. Because of these disadvantages, developing sustainable and cleaner energy forms is a global necessity and has become an urgent pursuit in scientific research.

Biodiesel is a renewable energy form that can be used as a substitute for fossil fuels. It is considered to be one of the best candidates to replace fossil diesel, because it can be directly used in any compression ignition engine without major modifications [4]. Biodiesel can be used in its pure form, but due to its high production cost and poor cold flow properties, it is often used as a diesel additive. Commercially used biodiesel is primarily composed of fatty acid methyl esters (FAMEs) that are produced via transesterification of triglycerides obtained from plants, animal fats, and microalgae [5].

Among all of the biodiesel production feedstocks, microalgae are considered to be the most promising feedstock due to their high rate and efficiency of photosynthesis. Microalgae can accumulate high lipid content in their biomass (up to 77% of total biomass), depending on the type of microalgae [6]. Compared to other available feedstocks for biodiesel production, microalgae have higher oil productivity and require less land area for cultivation. Most importantly, commercial-scale microalgae farms would not be in competition with food production [5].

Nevertheless, the main drawback of microalgae as a feedstock for biodiesel production is the energy intensive and costly production process, due mainly to the harvesting and the lipid extraction processes [7].

It is therefore necessary to develop effective methods to reduce the cost of these processes. For lipid extraction, two methods are often used: organic solvent extraction and supercritical CO_2_ (SCCO_2_) extraction. Organic solvent extraction requires a large volume of expensive and toxic organic solvents. It also requires an energy intensive lipid-solvent separation process after extraction, and not all of the solvent can be recycled. In addition, the lipid extraction rate is slow [8].

Compared to organic solvent extraction, SCCO_2_ extraction has several advantages. SCCO_2_ extraction is faster and has a higher selectivity toward biodiesel desirable lipid fractions, and the solvent-liquid separation process is not required, since SCCO_2_ will become gas after pressure is reduced [8]. The main drawback of SCCO_2_ extraction is its high equipment installation cost and its high energy requirement [9].

Previous researcher has demonstrated effective results for SCCO_2_ lipid extraction from microalgae. Halim et al. [10] studied the effect of SCCO_2_ on extracting lipid from the marine microalga* Chlorococcum *sp. They found that 80 min of SCCO_2_ extraction at 4400 psi and 30°C resulted in a maximum lipid yield of 7.1 wt% of dry biomass, which was equivalent to 5.5 h of hexane extraction using a Soxhlet apparatus. Mendes et al. [11] studied the SCCO_2_ lipid extraction of* Arthrospira maxima* with the addition of ethanol. They found that the addition of a polar modifier such as ethanol could greatly improve the results of SCCO_2_ extraction. Under the optimal experiment conditions of 60°C and 5000 psi, the addition of ethanol could increase the *γ*-linolenic acid (GLA) extraction yield from 0.05 wt% of dry biomass to 0.44%. Other researchers also have reported high efficiency supercritical CO_2_ extraction [12–14].

However, many of these experiments were conducted at the bench scale, and few of them used microalgae masses beyond 200 g. For a larger amount of microalgae sample, the effectiveness of SCCO_2_ extraction is not well investigated. Moreover, most of the SCCO_2_ extraction experiments were conducted at high pressures (at or above 5000 psi). Operating at such high pressures at commercial scale is more costly and could be potentially dangerous. Further, adding a polar modifier to the SCCO_2_ extraction process has been demonstrated to improve extraction efficiency [11]. Lorenzen et al. [15] conducted an industrial scale lipid extraction on the microalga* Scenedesmus *sp. using SCCO_2_ at low pressure (1740 psi) without any modifier or cosolvent in the extraction process, resulting in a long extraction time requirement (9 h) for a high lipid extraction yield.

The goal of this study is to investigate the scaling up potential of SCCO_2_ extraction for microalgae. Our study focused on SCCO_2_ extraction at a larger scale (from 200 g to 1 kg) at moderate pressures (below 5000 psi) with a modifier (methanol) added. A comparison between SCCO_2_ extraction and Soxhlet extraction was also conducted. The fatty acid profile in the extracted lipids was analyzed in order to predict the quality of biodiesel produced. Finally, a kinetic model was established to analyze the effects of pressure, methanol concentration, and CO_2_ flow rate on the lipid extraction yield.

## 2. Methodology

### 2.1. Optimizing the Operating Pressure of SCCO_2_ Extraction

**Figure 1:**
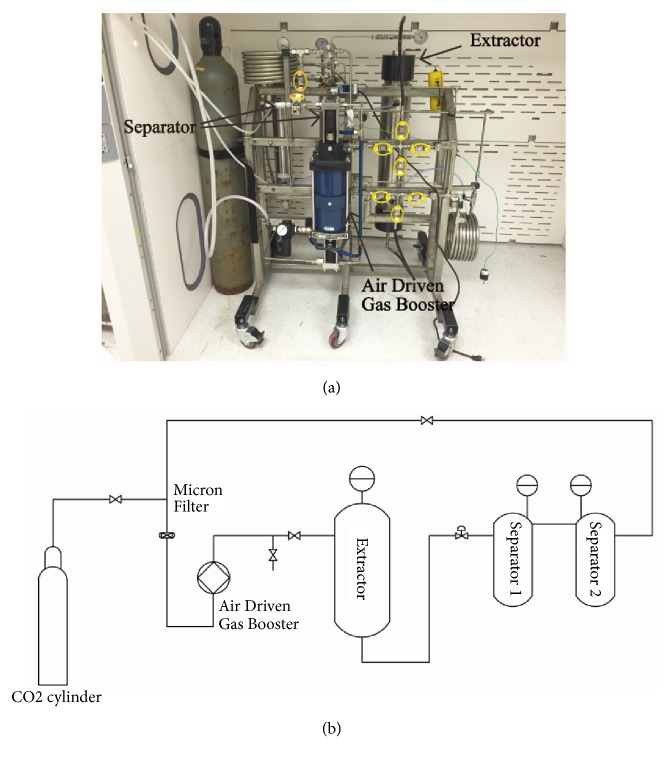
The supercritical CO_2_ extraction unit (a) and the schematic extraction process (b).

The SCCO_2_ extraction unit used in this research is shown in Figure 1(a).  Figure 1(b) is a schematic representation of the supercritical extraction process. 200 g of dry microalgae biomass is first placed in an extractor. An air-driven gas booster was used to pump CO_2_ from a cylinder to the extractor and increased the pressure in the extractor. The operating pressure has a significant impact on the SCCO_2_ extraction process, because the solubility of SCCO_2_ is a function of the pressure.

A pressure regulator was installed at the exit of the extractor in order to reduce the pressure in the separators, where the pressure was kept at 350 psi. The CO_2_ became a supercritical fluid and was able to extract lipids from the microalgae in the extractor, returned to a gas phase in the separators, and then was pumped back to the extractor by the air-driven gas booster. The extracted lipids were automatically collected in the separators. A valve was installed at the bottom of each separator to collect lipids after the operation. After the extraction process, the CO_2_ in the system was pumped back to the CO_2_ cylinder for recycling. Although two separators were used in the process, most of extracted lipids were collected in the first separator. The purpose of the second separator was to provide extra retention time to ensure pure CO_2_ was pumped back to the CO_2_ cylinder. The entire system was maintained at 50°C.

In order to investigate the effect of pressures, three different operating pressures were tested during the SCCO_2_ extraction process: 2000 psi, 3000 psi, and 4000 psi. Since the CO_2_ flow in the system was controlled by the air-driven gas booster, the pressure in the extractor also affected the CO_2_ flow rate in the system. The corresponding CO_2_ flow rates for the gas booster operated under 2000 psi, 3000 psi, and 4000 psi were 7.5, 7.1, and 6.8 standard cubic feet per minute (scfm), respectively, based on the performance curves of the gas booster. The residence time of CO_2_ in the extractor can be roughly estimated using the ratio of CO_2_ mass in the 5 L extractor to the CO_2_ mass flow rate. Under 2000 psi, 3000 psi, and 4000 psi of pressure, the residence times of CO_2_ were calculated as 0.13, 0.18, and 0.20 h, respectively.

Two different species of microalgae were tested in the SCCO_2_ extraction.* Spirulina *sp., a blue green alga commonly used as a food supplement or animal nutrition [16] with a lipid content of less than 33.3% dry weight of biomass (as reported by the producer), was purchased from Ojio® (India). The other species was* Schizochytrium *sp., which is a marine microalga characterized by its high content of polyunsaturated fatty acids [14], had a lipid content of 25% dry weight of biomass (as reported by the producer), and was purchased from Xiamen Huison Biotech Co. (China). The lipid content of this microalga was determined after docosahexaenoic acid (DHA) extraction conducted by the company. Both microalgae were obtained as dry powders and were used directly in the extraction without pre-treatment.

CHNS analysis and TGA analysis were conducted for both species to determine the elemental composition in microalgae biomass. For* Spirulina *sp., the composition was 45.83 wt% C, 6.52 wt% H, 10.73 wt% N, 0.66 wt% S, 20.76 wt% O, 6.46 wt% moisture, and 9.04 wt% ash. For* Schizochytrium *sp., the composition was 62.57 wt% C, 9.55 wt% H, 0.82 wt% N, 0 wt% S, 22.89 wt% O, 0.48 wt% moisture, and 3.69 wt% ash. These results were generally consistent with published data [17, 18]. Lower nitrogen and sulfur contents in biomass are preferred, since the biodiesel produced by that biomass will have lower exhaust emissions (such as NO_x_ and SO_2_) when used in a diesel engine [17]. Lower oxygen percentage in biomass is also desirable, since it can affect the stability of the produced biodiesel [17].

The SCCO_2_ extraction experiments were operated for 2, 4, and 6 h, and the extraction yield was measured after each time period during operation. The lipid extraction yield was calculated using the mass loss of the dried biomass before and after extraction, using the following equation:

(1) $$\begin{array}{c} Lipid\;\;extraction\;\;yield=\frac{W_{i}-W_{f}}{W_{i}} \end{array}$$

where *W*_i_ is the initial weight of the dried biomass (g) and *W*_f_ is the final weight of the dried biomass (g). The equation could overestimate the lipid extraction yield, since nonlipid components such as chlorophyll could also be extracted; however the equation was considered to be appropriate to compare the effectiveness of the extraction method for the tested conditions. Due to the physical characteristics of the extraction system, extracted lipids remaining in the extractor could not be easily measured; therefore the total mass of lipids was not used in the evaluation.

For comparison, lipid extraction using the organic solvent method was also investigated. A Soxhlet apparatus was charged with 25 g dry biomass, and 150 mL of methylene chloride/methanol (2:1, v/v) was refluxed for 6 h [19]. The extraction yield was calculated according to (1). An independent samples* t*-test was conducted to determine whether the difference was significant for the two extraction methods.

### 2.2. Investigating the Addition of Methanol and Its Effect on SCCO_2_ Extraction

Because SCCO_2_ can be treated as a nonpolar solvent, it will be difficult for SCCO_2_ to extract lipid complexes that are linked to the proteins on the microalgae cell membrane. Therefore, mixing SCCO_2_ with a polar solvent such as methanol might be a good way to increase the efficiency of SCCO_2_ extraction [20]. Methanol was chosen instead of ethanol due to its relative inexpensiveness, lower boiling point, and good solvation potential for polar components.

To investigate the effect of adding methanol to the SCCO_2_ extraction, 200 mL of methanol was added to the 5 L extractor before the extraction, so that the volume ratio of methanol/SCCO_2_ was 1:25 v/v. Experiments were conducted on both species mentioned previously. A stepwise addition of methanol was also tested, where 200 mL of methanol was added at 0, 2, and 4 h, for a total addition of 600 mL. The operating pressure used was similar to the previous experiments. The lipid extraction yield was measured and compared with SCCO_2_ extraction without the addition of methanol.

### 2.3. Investigating the Biomass Loading and Its Effect on SCCO_2_ Extraction

As stated previously, many experiments in the literature focused on small quantities of microalgae biomass. To assess the potential of scaling up the extraction process, the mass of microalgae in the extractor was increased from 200 g to 1 kg, while maintaining the porosity and the surface area. SCCO_2_ extraction was evaluated to see if similar lipid extraction yields could be achieved with larger biomass loading rates. The operating pressures and the amount of methanol added in the extractor were similar to those in the previous experiments. The residence time of CO_2_ in the extractor was kept constant when the biomass increased (see Section 2.1). An independent samples* t*-test was conducted to determine whether the difference was significant for different biomass loadings.

### 2.4. Characterization of the Extracted Lipids

The fatty acid profile of lipids in microalgae can affect the quality of biodiesel produced from microalgae lipids. Normally, cis-unsaturated fatty acids are favored over saturated fatty acids because the FAMEs derived from cis-unsaturated fatty acids often have advantageous cold flow properties [21]. The fatty acid profiles of the lipids from the two species were reported previously in published data [22, 23]. Therefore, matching the fatty acid profile in the extracted lipids and comparing to reported data in the literature can be a good indicator of the lipid extraction effectiveness of SCCO_2_.

The extracted lipids from the two species were characterized using gas chromatography (GC). Before the characterization, a derivatization step was conducted to convert the different compounds in the lipids to FAMEs. 1 mL of extracted lipid sample was collected in a test tube, and 1 mL of toluene and 2 mL of 1% sulfuric acid in methanol were added into the test tube. This test tube was then sealed and stored in a 50°C oven for 8 h. After cooling, 5 mL of 5% sodium chloride in water was added to the test tube to encourage phase separation. After phase separation, 1 mL of the top organic phase was collected in a GC vial for analysis. An Agilent 7890B GC containing a DB-23 column (60 m length and 0.15 *μ*m film) and a flame ionization detector was used with helium as the carrier gas. The FAME mixture (C8-C24) (Sigma Aldrich) was used as the calibration standard with methyl myristate as the internal standard. The operating conditions of the GC system were selected according to the literature [24].

### 2.5. Kinetic Study of the SCCO_2_ Extraction

The lipid extraction from microalgae using SCCO_2_ can be expressed using first-order kinetic as follows [10]:

(2) $$\begin{array}{c} M_{e}=M_{i}\left(\;1-e^{-kt}\;\right) \end{array}$$

where* M*_*e*_ is the amount of extracted lipids at time* t *(g/g biomass),* M*_*i*_ is the total lipid content in microalgae cell (g/g biomass),* t *is the extraction time (h), and* k* (h^−1^) is a time constant correlated to the lipid mass transfer from the microalgae cells to SCCO_2_.

For a typical mass transfer problem, the time constant* k* is a function of Reynolds number (*Re*) and Schmitt number (*Sc*), as shown in the following equation:

(3) $$\begin{array}{c} k=F\left(\;Re,Sc\;\right) \end{array}$$

In SCCO_2_ extraction, the values of the dimensionless numbers* Re* and* Sc* are dependent on pressure, temperature, concentration of methanol, and CO_2_ flow rate. Therefore, the time constant* k* can be expressed as

(4) $$\begin{array}{c} k=F\left(\;Re,Sc\;\right)=f\left(\;P,T,\left[\;ME\;\right],Q\;\right) \end{array}$$

where* P* is pressure (psi),* T* is temperature (°C), [*ME*] is the concentration of methanol in SCCO_2_ (v/v), and* Q* is the flow rate of CO_2_ (scfm).

To simplify the model, the effect of temperature was eliminated in (4) due to the constant temperature used in this research. The extracted lipids were treated as a single substance instead of a compound. It was assumed that the all of the extractable lipids could be extracted eventually; therefore all of curves under different operating conditions will have the same asymptotes.* M*_*i*_ of the two microalgae species was assumed to be 0.25 g/g biomass. In addition, the methanol concentration in SCCO_2_ was assumed to be constant (4%) during the extraction process. Also, the methanol concentration during the stepwise addition was also assumed to be constant (4%). To study the effects of* P, *[*ME*], and* Q* on the time constant* k* and lipid extraction yield, we assumed a first-order equation to express the relation:

(5) $$\begin{array}{c} k=a*P+b*\left[\;ME\;\right]+c*Q+d \end{array}$$

where* a, b, c, d* are unknown constants. These four constants can be estimated by fitting the kinetic model with the experimental data obtained from the previous sections. MATLAB programming platform was used to perform the model fitting, and these constants were determined by minimizing the sum of squared errors between experimental and calculated values of lipid extraction yield. The minimization was performed using the MATLAB toolbox fmincon. Since the lipid mass transfer coefficients may be different for different microalgae species, the estimation of the constants was conducted separately for the two species.

To validate the kinetic model, SCCO_2_ extraction data obtained by a previous research conducted by Nobre et al. [25] was also modeled. The authors of the research investigated the lipid extraction yield from microalga* Nannochloropsis *sp. under different pressures, flow rates, and ethanol concentrations in a laboratory scale SCCO_2_ extraction system (5 cm^3^ extraction vessel). To perform the model fitting, the ethanol concentration was used in place of [*ME*] (methanol) in (5). The data used for parameter fitting included the lipid extraction yields at 40°C, pressures of 1800, 2900, or 4350 psi, CO_2_ flow rates of 6.64×10^−3^ or 1.18 ×10^−2^ scfm, and ethanol concentrations of 5, 10, or 20 wt%.* M*_*i*_ used for* Nannochloropsis *sp. was 0.45 g/g biomass, based on the reported results by the authors, and the biomass loading of* Nannochloropsis *sp. was 1.25 g.

After obtaining the constants, a sensitivity analysis was conducted to analyze the influence of* P, *[*ME*], and* Q *on the simulated results. Sensitivity analysis was carried out by calculating the lipid mass transfer coefficient (*k*) and lipid extraction yield at 4 h under the conditions where the value of one parameter was changed by 50% without changing other parameters. The experiment conditions used in the sensitivity analysis were 4000 psi of pressure, 200 mL of methanol with stepwise addition, and 6.8 scfm of CO_2_ flow rate. For microalga* Nannochloropsis *sp., the experiment conditions used were 4350 psi of pressure, 1.18×10^−2^ scfm of CO_2_ flow rate, and 5 wt% of ethanol concentrations [25].

## 3. Results 

### 3.1. Lipid Extraction Yields of SCCO_2_ under Different Operating Pressures

**Figure 2:**
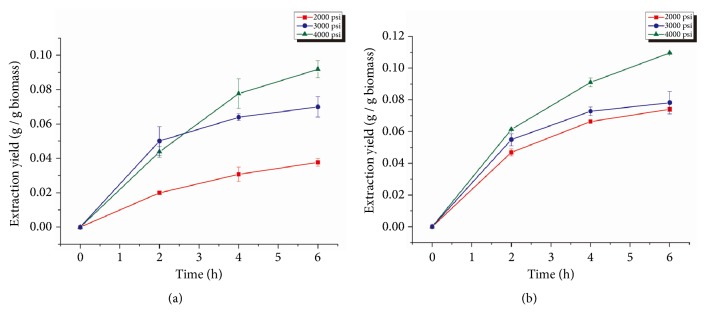
Lipid extraction yield from* Spirulina *sp. (a) and* Schizochytrium *sp. (b) using SCCO_2_ under different operating pressures. Error bars represent one standard error from three replicates.

Different operating pressures of SCCO_2_ resulted in a significant difference in lipid extraction effectiveness from* Spirulina *sp. (Figure 2(a)). As operating pressures increased, the extraction yield increased, and 4000 psi SCCO_2_ resulted in the highest lipid extraction yield. A similar trend was observed in SCCO_2_ extraction from* Schizochytrium *sp. (Figure 2(b)). These results suggest that higher operating pressures should be selected when conducting SCCO_2_ extraction; therefore 4000 psi was chosen as the operating pressure for subsequent experiments.

### 3.2. Lipid Extraction Yields of SCCO_2_ with Addition of Methanol

**Figure 3:**
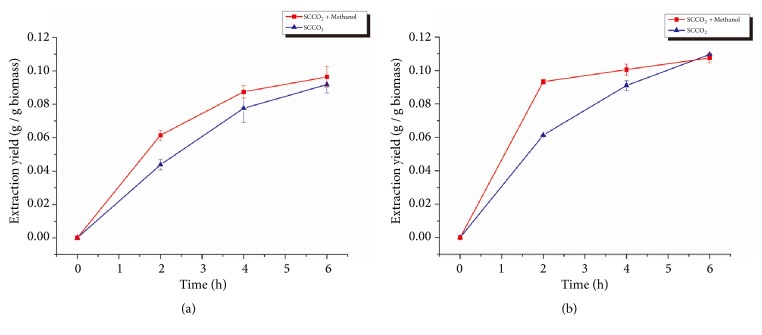
Lipid extraction yield from* Spirulina *sp. (a) and* Schizochytrium *sp. (b) using SCCO_2_ with a single initial methanol addition. Error bars represent one standard error from three replicates.

As shown in Figure 3, adding 200 mL of methanol as a cosolvent increased the lipid extraction yield of SCCO_2_ for both species in the first 2 h. However, after the initial 2 h, the effectiveness of extraction due to the added methanol decreased and eventually became insignificant after 4 h of extraction. These results indicated that the methanol added to the SCCO_2_ extractor might be washed away with the SCCO_2_ flow during the extraction process resulting in no increase in lipid extraction yield observed after 6 h of extraction. These results suggested that adding methanol once may not be a good method. To examine this possibility, a stepwise addition of methanol was applied in subsequent experiments.

**Figure 4:**
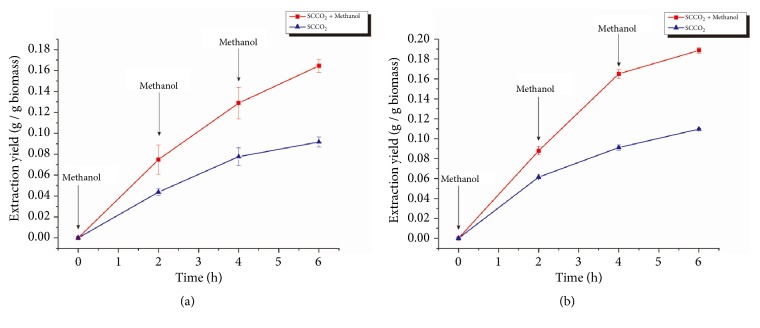
Lipid extraction yield from* Spirulina *sp. (a) and* Schizochytrium *sp. (b) using SCCO_2_ with a stepwise addition of methanol. Error bars represent one standard error from three replicates.

SCCO_2_ lipid extraction with a stepwise addition of methanol was also tested by adding 200 mL of methanol every 2 h into the extractor to keep the methanol concentration in SCCO_2_ at 4% (v/v), as shown in Figure 4. The stepwise addition of methanol significantly increased the lipid extraction yield for both species compared with pure SCCO_2_ extraction. An 80% increase in lipid extraction yield was found for* Spirulina *sp., while a 72% increase was found for* Schizochytrium *sp. These results suggest that the stepwise addition of methanol overcame the decreasing methanol concentration and was more effective than adding methanol once. Average yields of 0.164 ± 0.006 and 0.188 ± 0.003 were obtained after 6 h of SCCO_2_ extraction with stepwise methanol addition for* Spirulina* sp. and* Schizochytrium *sp., respectively.

### 3.3. Soxhlet Lipid Extraction Using Organic Solvents

**Figure 5:**
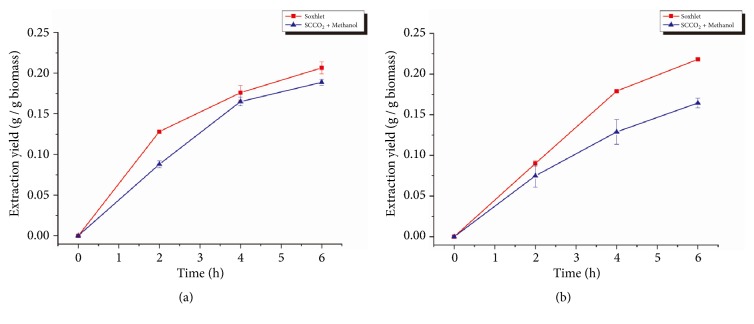
Comparison of lipid extraction yield from* Schizochytrium *sp. (a) and* Spirulina *sp. (b) using Soxhlet extraction and SCCO_2_ extraction. Error bars represent one standard error from three replicates.

The Soxhlet extraction of lipids was conducted for both microorganisms and compared with the highest extraction yield obtained from the SCCO_2_ extraction, which was achieved at 4000 psi of SCCO_2_ with methanol added sequentially (Figure 5). The Soxhlet extraction resulted in higher lipid extraction yield for both species. For* Schizochytrium *sp., the methanol-SCCO_2_ extraction produced 91.4% of the lipid extraction yield obtained from Soxhlet extraction after 6 h, but the difference was not statistically significant. For* Spirulina* sp., the methanol-SCCO_2_ extraction produced 79.6% of the lipid extraction yield obtained from Soxhlet extraction, which was statistically significant. While these results indicate that methanol-SCCO_2_ extraction is not superior to Soxhlet extraction, the methanol-SCCO_2_ method demonstrated comparable extraction yield while avoiding the use of a toxic solvent such as methylene chloride.

### 3.4. Investigating the Scale-Up Potential

**Figure 6:**
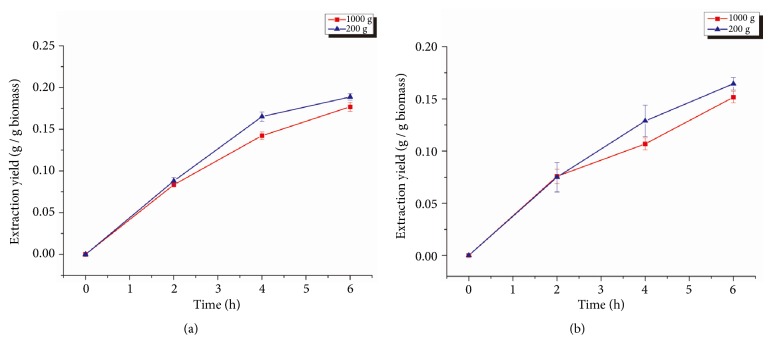
Lipid extraction yield from* Schizochytrium *sp. (a) and* Spirulina *sp. (b) using methanol-SCCO_2_ with different biomass loadings. Error bars represent one standard error from three replicates.

To investigate the scale-up potential of methanol-SCCO_2_ extraction, experiments were conducted with increased biomass loadings of up to 1 kg while maintaining other operating conditions (4000 psi, 200 mL methanol added sequentially every 2 h). As shown in Figure 6, increasing the biomass loading from 200 g to 1 kg resulted in a slight decrease in lipid extraction yields for both species. The lipid extraction yield for* Schizochytrium* sp. decreased by 7.4%, while lipid extraction yield for* Spirulina *sp. decreased by 7.8%, although neither difference was statistically different. These results demonstrated that the loading amount of microalgae biomass could be increased in SCCO_2_ extraction without a significant loss of extraction yield.

### 3.5. Characterizing the Fatty Acid Profile in Extracted Lipids

For* Spirulina* sp., the fatty acids extracted by the Soxhlet method differed significantly from those extracted by SCCO_2_, particularly in the composition of decanoic acid (C10), palmitic acid (C16), and total unsaturated fatty acids (Table 1). The total unsaturated fatty acids were the sum of myristoleic acid (C14:1), palmitoleic acid (C16:1), oleic acid (cis C18:1), linoleic acid (cis C18:2), and linolenic acid (C18:3). On the other hand, the methanol-SCCO_2_ extraction resulted in a similar fatty acid composition to the Soxhlet method. The Soxhlet method was presumed to be most representative of complete lipid extraction. The fatty acid compounds were mostly C16 and C18, while total of 47.25% of unsaturated fatty acids was found in the extracted lipids.

The SCCO_2_ method extracted significantly lower amounts of unsaturated fatty acids compared to the Soxhlet method, while greatly increasing the content of C10 compounds. However, when combining SCCO_2_ with methanol, the fatty acid profile of the extracted lipids was similar to that obtained by the Soxhlet method, with C16 and C18 as the main compounds and total unsaturated fatty acids of 45.11%. These results indicate the content of unsaturated fatty acids in extracted lipids was greatly affected by the polarity of the extraction fluid in that increasing the polarity of the extraction fluid increased the total unsaturated fatty acids extracted.

For* Schizochytrium* sp., it can be observed that the three extraction methods used in this research resulted in similar fatty acid compositions with no significant differences (Table 2). Unlike* Spirulina* sp., no unsaturated fatty acids were found in* Schizochytrium* sp.; therefore the difference induced by the polarity of extraction fluid became insignificant, which resulted in the similar fatty acid profiles.

### 3.6. Kinetic Study of the SCCO_2_ Extraction

**Figure 7:**
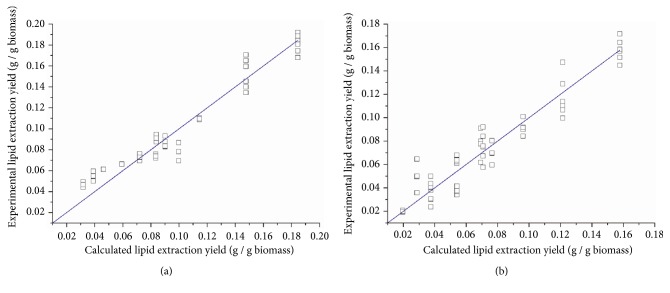
Parity plot of calculated lipid extraction yields versus experimental lipid extraction yields for all experiments. (a)* Schizochytrium *sp. (b)* Spirulina *sp.

The estimated values of* a, b, c, *and* d* after model fitting with MATLAB are shown in Table 3. The parity plots of calculated lipid extraction yields versus experimental lipid extraction yields for all experiments in this research are presented in Figure 7. For* Schizochytrium* sp., an average error of 12.7% was found between the experimental data and calculated values while for* Spirulina *sp., the average error was 16.6%.

**Figure 8:**
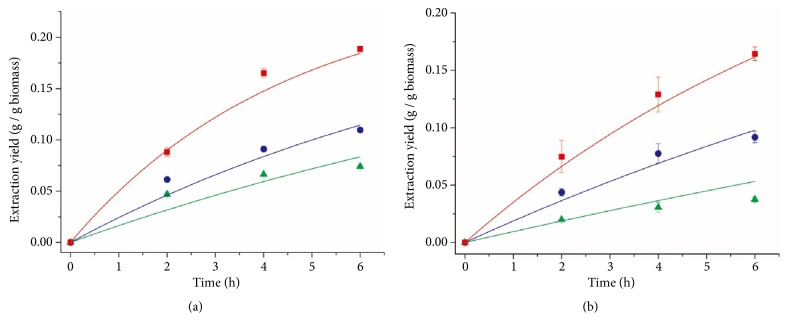
Comparison of simulated lipid extraction yields (lines) and experimental lipid extraction yields (symbols) under different operating pressures and the test when methanol was added. (a)* Schizochytrium* sp.: red square, 4000 psi with 200 ml of methanol, r^2^ = 0.985; blue circle, 4000 psi, r^2^ = 0.957; green triangle, 2000 psi, r^2^ = 0.885. (b)* Spirulina* sp.: red square, 4000 psi with 200 ml of methanol, r^2^ = 0.992; blue circle, 4000 psi, r^2^ = 0.974; green triangle, 2000 psi, r^2^ = 0.608. Error bars represent one standard error from three replicates.

Figure 8 shows the comparison of simulated and experimental lipid extraction yields at 2000 and 4000 psi, as well as 4000 psi with the addition of methanol. Generally good agreement was observed for both species. However, for* Spirulina *sp., the simulated results were more accurate under high operating pressures; therefore 4000 psi was chosen as the operating pressure for sensitivity analysis.

**Figure 9:**
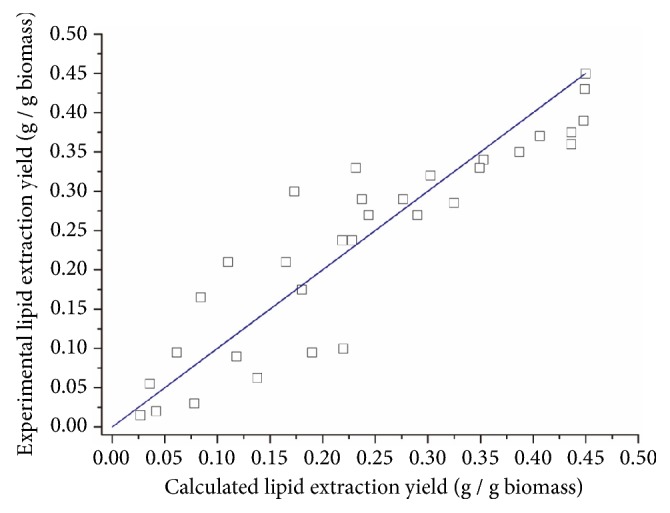
Parity plot of calculated lipid extraction yields versus experimental lipid extraction yields using the data of SCCO_2_ extraction from microalga* Nannochloropsis* sp. [25].

**Figure 10:**
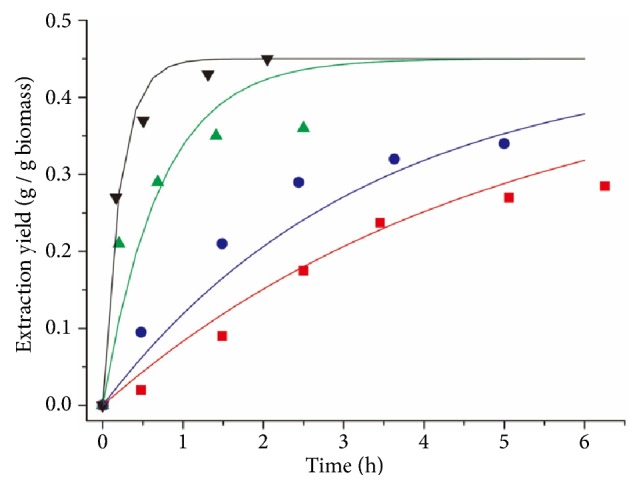
Comparison of simulated lipid extraction yields (lines) and experimental lipid extraction yields (symbols) from microalga* Nannochloropsis* sp. [25] under different conditions. Red square, 2900 psi of pressure, 6.64×10^−3^ scfm of CO_2_ flow rate, r^2^ = 0.960; blue circle, 4350 psi of pressure, 6.64×10^−3^ scfm of CO_2_ flow rate, r^2^ = 0.931; green triangle, 4350 psi of pressure, 1.18×10^−2^ scfm of CO_2_ flow rate, 5 wt% of ethanol concentration, r^2^ = 0.802; black inverted triangle, 4350 psi of pressure, 1.18×10^−2^ scfm of CO_2_ flow rate, 20 wt% of ethanol concentration, r^2^ = 0.982.

Applying the kinetic model to the results from Nobre et al. [25], the estimated values of* a, b, c, *and* d* are shown in Table 3. The parity plot of calculated lipid extraction yields versus experimental lipid extraction yields (Figure 9) showed an average error of 35.6%, indicating the model was less accurate for the SCCO_2_ extraction of* Nannochloropsis *sp. However, model agreement improved between simulated values and experimental data when the extraction was conducted at high operating pressures (Figure 10).

Results of the sensitivity analysis suggest that the influences of pressure, methanol concentration, and CO_2_ flow rate on lipid extraction yield were species dependent (Table 4). However, for all species the methanol concentration (or ethanol concentration) in SCCO_2_ had the strongest influence on the lipid extraction yield, followed by the operating pressure. While the CO_2_ flow rate showed some influence on the lipid extraction yield for* Schizochytrium* sp., it was insignificant for* Spirulina *sp. and* Nannochloropsis *sp.

## 4. Discussion 

In our study, results showed that the effectiveness of SCCO_2_ lipids extraction increased with an increasing pressure for both species. This is an expected result and matches the findings of the work published by other researchers [26]. It is generally believed that, at a constant temperature, increasing the pressure increases the density of SCCO_2_, which increases the solubility of lipids [8]. Thus, a higher operating pressure of SCCO_2_ would produce a stronger lipid extraction power. Therefore, the highest operating pressure that the equipment could sustain, which was 4000 psi, was chosen as the optimal operating pressure for subsequent experiments.

The effect of methanol as a cosolvent in SCCO_2_ extraction was shown to increase the lipid extraction yield at least for the first 2 h. The reason for the improved extraction yield can be explained by the polarity of CO_2_ and methanol. The CO_2_ molecule is a nonpolar molecule and can attach to nonpolar acylglycerols in microalgae cytoplasm by Van der Waals forces during the extraction process [27]. However, some of the acylglycerols in microalgae were associated with proteins on cell membrane via hydrogen bonds. The Van der Waals forces were not strong enough to disrupt the hydrogen bonds. On the other hand, methanol molecules are polar and can easily form hydrogen bonds with the proteins on the cell membrane and replace those lipids, leaving the lipids to be extracted by SCCO_2_ [27] thus increasing lipid extraction yield when methanol was added.

Nevertheless, the effectiveness of the methanol addition decreased over time. It was assumed that the methanol added to the SCCO_2_ extractor was washed away with the SCCO_2_ flow during the extraction process, since methanol was easily soluble in SCCO_2_. Because the SCCO_2_ would return to gas status in the separator, methanol would accumulate in the separator, which led to the reduction of methanol concentration in the extractor. In order to overcome this problem, a stepwise addition of methanol was applied for the SCCO_2_ extraction, and the results indicated that adding methanol sequentially to the SCCO_2_ extractor resulted in higher extraction efficiency compared to adding methanol at once.

Soxhlet extraction using methylene chloride/methanol (2:1 v/v) was reported to be a superior effective lipid extraction method and a good substitute for the Bligh and Dyer method for its lower toxicity. The lipid extraction yields after 6 h of Soxhlet extraction reached 0.21 g/g biomass and 0.22 g/g biomass for* Spirulina *sp. and* Schizochytrium* sp. These results were consistent with the reported lipid content on the label (below 33 wt% for* Spirulina *sp. and 25 wt% for* Schizochytrium* sp.). The methanol-SCCO_2_ extraction method was not significantly different from, or only slightly less effective than, the Soxhlet extraction. Further, the methanol-SCCO_2_ extraction excelled in its low toxicity and requirement of organic solvent, since only 600 mL of methanol was used for a 5 L extraction chamber, which can be loaded with 1 kg of biomass (0.6 mL/g). By comparison, the Soxhlet extraction required 100 mL of methylene chloride and 50 mL of methanol for every 25 g of biomass (6 mL/g). Methylene chloride usage, which is considered carcinogenic, was also avoided. Overall, the results indicated that the methanol-SCCO_2_ extraction method can produce high lipid extraction yield and might be a good replacement for the organic solvent extraction. In addition, further improvements could be considered to increase the efficiency of the methanol-SCCO_2_ extraction method, such as optimizing the methanol amount added at each stage of stepwise addition, or optimizing the temperature provided for the extraction system. Increasing pressure could also be an option, although it may also increase the capital and operating expenses.

The effect of biomass loading rate was also investigated, and it was found that a statistically insignificant decrease in lipid extraction yield occurred when the biomass loading rate was increased from 200 g to 1 kg. The decrease in yield may be explained by the fact that increasing the biomass loading increases the packing density of the microalgae. This might hinder overall extraction as the lipids may reabsorb on the microalgae surface or cause nonhomogeneous extraction via fluid channeling effects [28]. However, the decrease in yield was less than 8% for both species implying that scale-up can be successful and that the methanol-SCCO_2_ extraction method is feasible for 1 kg biomass loadings with our apparatus.

For* Spirulina* sp., the fatty acid compositions obtained from the Soxhlet and methanol-SCCO_2_ method supported published data [22, 29], while the SCCO_2_ extraction without methanol showed a significant decrease in the extraction of palmitic acid and total unsaturated fatty acids. These results indicate that using SCCO_2_ alone was insufficient in obtaining the ideal fatty acid composition from* Spirulina *sp. due to its ineffectiveness in targeting the lipids associated with cell membrane proteins. Adding methanol to SCCO_2_ increased the extracting power of SCCO_2_ by extracting all types of fatty acids in the cells. For the* Schizochytrium *sp., the three extraction methods did not show significant differences. These results also supported published data except for the composition of DHA [23, 30]. Since the microalgae used in these experiments were obtained after a DHA extraction process, the absence of DHA was expected. In general, methanol-SCCO_2_ extraction could recover lipids with fatty acid composition similar to that extracted by the Soxhlet extraction, proving the effectiveness of this method.

In the kinetic study of the SCCO_2_ extraction, the estimated values of* a, b, c*, and* d* varied greatly as a function of species and operating parameters. However, the sensitivity analysis of the kinetic modeling showed similar results for all species, indicating that both the operating pressure and concentration of methanol (or ethanol) in SCCO_2_ greatly affected the lipid extraction yield. Therefore, to increase lipid extraction yield, the addition of a polar modifier (such as methanol) in the SCCO_2_ process might be preferred to increasing pressure, since the increase in pressure would also increase the operating cost. On the other hand, it is not recommended to increase the CO_2_ flow rate since it had little or no influence on the lipid extraction yield and the mass transfer coefficient. Similar results were also found by Nobre et al. [25] where the authors noted that the flow rate of CO_2_ had no influence on lipid extraction yield.

## 5. Conclusions 

SCCO_2_ combined with methanol demonstrated a high efficiency in lipid extraction from microalgae, as well as a high potential for process scale-up. The high lipid extraction yield and the fatty acid composition in the extracted lipids indicated that the methanol-SCCO_2_ extraction method can be used as a good substitute for the Soxhlet extraction method, while the requirement of organic solvents was significantly reduced. At same time, the lack of usage of toxic organic solvents such as methyl chloride showed that the methanol-SCCO_2_ extraction was safer than organic solvent extraction, suggesting it might be a preferable method in commercial-scale extraction. It should be noted that dry biomass was used in this research. Since the drying process is typically energy intensive, it would be preferable if wet biomass was used in the extraction process. Future research involving the extraction of wet biomass is needed. In addition, further studies regarding the optimization of the rate of methanol addition during the extraction process, as well as temperature effects, should be investigated in order to optimize both the extraction efficiency and economic feasibility of supercritical CO_2_ lipid extraction from microalgae.

## Figures and Tables

**Table 1:** Fatty acid profile of extracted lipids from *Spirulina *sp. using three extraction methods.

**Fatty acid Compounds**	**wt**%		
	**Soxhlet**	**SCCO**_**2**_	**SCCO**_**2**_ ** + methanol**
C10	7.48 ± 0.06	47.21 ± 0.24	16.51 ± 0.10
C14:1	1.79 ± 0.01	-	-
C16	44.02 ± 0.23	22.41 ± 0.14	37.15 ± 0.31
C16:1	2.22 ± 0.01	1.97 ± 0.01	2.66 ± 0.01
C18	1.25 ± 0.01	1.57 ± 0.01	1.23 ± 0.01
Cis C18:1	2.65 ± 0.03	2.85 ± 0.01	3.38 ± 0.01
Cis C18:2	18.10 ± 0.34	12.27 ± 0.06	17.67 ± 0.28
C18:3 n6	18.26 ± 0.12	9.43 ± 0.03	18.44 ± 0.09
C18:3 n3	4.23 ± 0.10	2.29 ± 0.01	2.96 ± 0.24

Total unsaturated fatty acids	47.25	28.81	45.11

**Table 2:** Fatty acid profile of extracted lipids from *Schizochytrium* sp. using three extraction methods.

**Fatty acid Compounds**	**wt**%		
	**Soxhlet**	**SCCO**_**2**_	**SCCO**_**2**_ ** + methanol**
C14	8.28 ± 0.01	8.30 ± 0.01	8.23 ± 0.02
C16	88.76 ± 0.01	87.05 ± 0.05	88.83 ± 0.04
C18	2.96 ± 0.01	4.65 ± 0.06	2.94 ± 0.05

Total unsaturated fatty acids	0	0	0

**Table 3:** The estimated values of *a, b, c, *and* d *after model fitting with MATLAB.

Parameters	*Schizochytrium*	*Spirulina*	*Nannochloropsis*
*a *(h^−1^ psi^−1^)	1.96 × 10^−5^	2.02 × 10^−5^	7.05 × 10^−5^
*b *(h^−1^)	2.98	2.13	21.7
*c *(h^−1^ scfm^−1^)	3.47 × 10^−3^	1.15 × 10^−5^	1.25 × 10^−4^
*d *(h^−1^)	8.01 × 10^−4^	9.51 × 10^−5^	4.59 × 10^−7^

**Table 4:** Sensitivity analysis of *P*, [*ME*], and *Q* on the simulated results.

	Parameter change	Deviation with parameter change (%)								
		*Schizochytrium*			*Spirulina*			*Nannochloropsis*		
		*P*	[*ME*]	*Q*	*P*	[*ME*]	*Q*	*P*	[*ME*]	*Q*
*k*	+50%	+17.69	+26.81	+5.32	+24.31	25.64	+0.02	+11.04	+38.96	+0
	-50%	-17.69	-26.81	-5.32	-24.31	-25.64	-0.02	-11.04	-38.96	+0

Lipid extraction yield	+50%	+10.16	+14.82	+3.22	+15.80	+16.60	+0.02	+0.18	+0.34	+0
	-50%	-11.89	-18.80	-3.38	-18.58	-19.69	-0.02	-0.33	-2.99	+0

## Data Availability

The figures and tables used to support the findings of this study are included within the article.
